# Double Aortic Contour in Chest Radiography

**DOI:** 10.7759/cureus.84188

**Published:** 2025-05-15

**Authors:** Thomas Saliba, David Rotzinger, Denis Tack

**Affiliations:** 1 Radiology, Centre Hospitalier Universitaire Vaudois (CHUV), Lausanne, CHE; 2 Radiology, Centre Hospitalier EpiCURA - site de Ath, Ath, BEL

**Keywords:** adenopathy, aorta, chest x-ray, double aortic contour, mediastinum

## Abstract

Mediastinal lymphadenopathy may manifest subtly on routine chest radiographs, particularly when arising in the left paraaortic region, where the normal pleural reflection across the aortic arch (“left paraaortic stripe”) can be obscured. We report the case of an 82-year-old man with a history of renal cell carcinoma in remission who presented with fatigue, mild dyspnea, and weight loss. A postero-anterior chest X-ray revealed a double aortic contour, absent on imaging two years prior, raising the suspicion of lymphadenopathy. This prompted a non-contrast CT that identified a 2 cm paraaortic lymph node metastasis. Surgical resection was successful, though the patient later died of unrelated causes. Recognition of a double aortic contour, created by an adjacent mass displacing mediastinal fat of differing attenuation, is crucial, yet detection is often hampered by variable pleural reflections and the small size or location of nodal disease. Comparative radiographs greatly enhance diagnostic confidence. However, given that fewer than half of aortopulmonary window lymphadenopathies are visible on X-ray and false negatives are common, CT confirmation remains essential when mediastinal lymphadenopathy is suspected.

## Introduction

Mediastinal lymphadenopathy is a common finding in a multitude of diseases, including neoplasms and granulomatous diseases [1]. The left paraaortic stripe, as described by Keats and Schwartz et al., is a clear space, which is normally present on routine posteroanterior chest radiographs, consisting of the descending aorta, pulmonary outflow tract, and left upper lobe vessels that form its medial, inferior, and lateral boundaries, respectively [1,2]. The medial edge of this space has a concave outer edge [2]. Several pathologies may widen this area, including cardiomegaly, pericardial effusion, aneurysms, mediastinal cysts or masses. Additionally, neural tumours or volume loss of the left upper lobe can alter the appearance of the paraaortic stripe [1].

Chest X-rays are commonly used as a first-line imaging method and thus may be the first type of imaging in which left paraaortic masses will be visualized.

Given the broad differential diagnoses, early detection of abnormalities on chest radiographs is crucial to appropriately guide subsequent diagnostic imaging. This will generally involve a chest CT scan, possibly with the addition of intravenous contrast.

## Case presentation

An 82-year-old man with a known history of renal cell carcinoma (RCC), initially diagnosed five years earlier, presented with progressive fatigue, mild dyspnea on exertion, and unexplained weight loss.

The patient has previously undergone a right nephrectomy and completed chemotherapy. The patient was subsequently classed as being in full remission with no evidence of recurrence on previous follow-up imaging up to this point.

**Figure 1:**
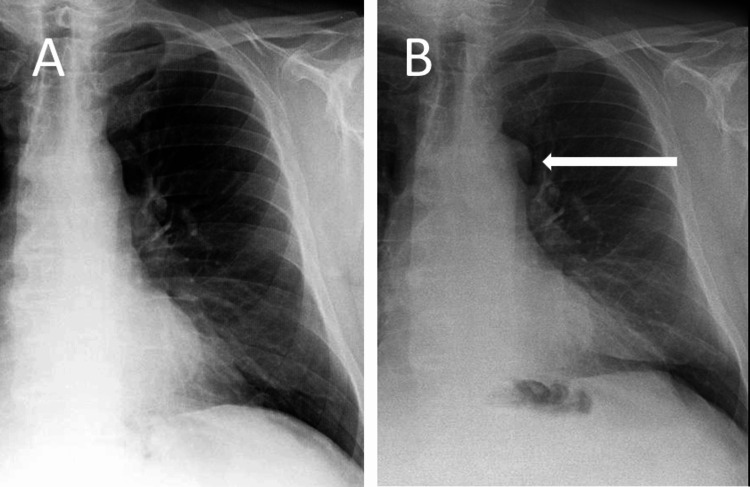
Posteroanterior chest radiographs of the patient at two different time points Panel A shows the normal chest radiograph of the patient without a double aortic contour. Panel B shows a double aortic contour (arrow) due to the lymphadenopathy.

**Figure 2:**
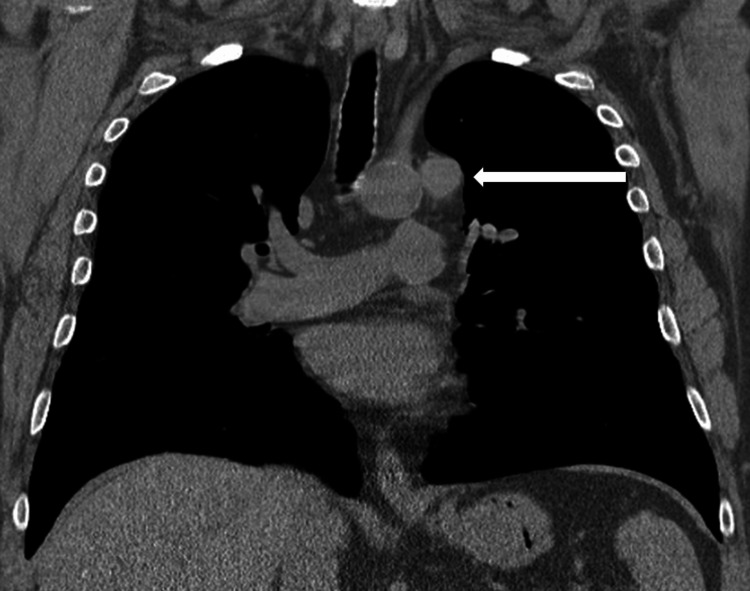
Coronal reformat of unenhanced chest CT imaging at the level of the trachea Coronal reformat of an unenhanced chest CT at the level of the trachea, showing a 2 cm lymphadenopathy located left to the aortic arch (arrow). One can also note the minor aortic arch calcifications.

A chest X-ray was requested to investigate the unexplained symptoms, with the presumptive diagnosis of infection or secondary malignancy. The postero-anterior chest X-ray revealed a soft tissue density anomaly to the left of the aortic arch. Fortunately, it was possible to compare this X-ray to a prior one obtained two years earlier, confirming that the mass, which resulted in the appearance of a double aortic contour (Figure 1), was not previously visible. This prompted the radiologist to suggest the presence of a mediastinal lesion, with the recommendation that a CT scan be performed to confirm the diagnosis. As the patient had a glomerular filtration rate of under 30 mL/min/1.73 m^2^, a non-contrast CT scan was performed in order to preserve the patient’s renal function (Figure 2), which revealed that the paraaortic mass corresponded to a 2 cm lymphadenopathy.

The patient underwent successful surgical resection of the lymph node metastasis but passed away from unrelated causes.

This case underlines the importance of subtle chest X-ray signs and how any anomalies should prompt further investigation, especially in the context of patients with a known history of cancer.

## Discussion

Epidemiology

Renal cell cancers are infrequent, only accounting for 3% of malignancies worldwide [3]. The lungs are the most common area of metastasis, accounting for 70.9% of cases, with lymph node metastasis being found in 37.9% of metastatic patients [3]. Patients who develop lymph node metastasis have an average survival time of 20 months [3].

Clinical relevance

Chest X-rays are commonly used in the workup of chest pathologies, regardless of whether the patient is known for having a pre-existing oncological condition. This makes the ability to recognize anomalies such as lymphadenopathy vital in patients for whom the differential diagnosis may be vast, as it may serve as the first sign of disease progression.

Radiographic features

A normal chest X-ray typically reveals a single aortic contour on the left side. However, when two distinct outlines are visible, this is referred to as a double aortic contour, suggesting the presence of an additional structure adjacent to the aorta, which can arise from anatomical variations or pathological changes such as aortic aneurysm, left upper lobe collapse, or pleural thickening. Furthermore, the variations in the pleural reflections in this area complicate the distinction between normality and pathology, thus making previous comparative exams essential in detecting changes [4]. The visibility of this double contour relies on the presence of the mass in addition to a displacement of adjacent mediastinal fat that differs in attenuation as compared to that of the aorta. The double aortic contour shown here cannot correspond to any intraluminal aortic disorder, such as dissection, because on a chest radiograph, the attenuation of the dissected layer is almost indistinguishable from that of the circulating blood. Furthermore, there were no other suggestive signs of dissection, such as the displacement of aortic calcifications [5].

This presentation is rare because the location of the corresponding lymphadenopathy is unusual, referring to level 6 in usual mediastinal maps [6,7]. Indeed, when a carcinoma develops in the left lung, the lymph node involvement is usually in the aortopulmonary window (referred to as 5L), and from this location, the disease progresses to the anterior mediastinum (a location referred to as 2L) or the middle mediastinum in the left paratracheal space (4L).

Finally, the detection of a double aortic contour may be difficult in two circumstances: first, if this sign is not known as a potential significant abnormality, and second, if the actual radiograph is not compared to an available prior one. Indeed, comparing radiographs is likely to provide a high certainty of disease when requesting a CT to confirm the presence or absence of a mass located next to the aortic arch. 

It should be noted, however, that the detection of lymphadenopathy around the aortopulmonary window is challenging, with only around half of the cases being visible on X-rays [8].

Differential diagnoses

The differential diagnosis for opacities in this region is vast and includes aneurysms, lymphadenopathy, primary tumours, metastasis, anatomical variants, or even post-operative changes [8].

Research has shown that the location of the mass is very important, as it should extend at least 1 cm beyond the aortic arch to be distinguishable; fulfilling this requirement is more important than the actual size of the mass [8].

## Conclusions

The appearance of a double aortic contour on chest X-rays may be useful in detecting lymphadenopathies in level 6 of the left mediastinum, particularly in patients with prior abdominal cancer disease. This is important as early detection could change the outcome of the patient, particularly in oncological cases. Nevertheless, due to the difficulty of detecting these lesions and the large proportion of false negatives, a CT scan should always be performed in cases of suspected mediastinal lymphadenopathy.

## References

[1] Schwarz ML, Marmorstein BL (1975). A radiographic sign of left sided mediastinal lymph node enlargement. Chest.

[2] Keats TE (1972). The aortic-pulmonary mediastinal stripe. Am J Roentgenol Radium Ther Nucl Med.

[3] Lee CH, Kang M, Kwak C (2024). Sites of metastasis and survival in metastatic renal cell carcinoma: results from the Korean Renal Cancer Study Group database. J Korean Med Sci.

[4] Blank N, Castellino RA (1972). Patterns of pleural reflections of the left superior mediastinum. Normal anatomy and distortions produced by adenopathy. Radiology.

[5] Gartland S, Sookur D, Lee H (2007). Aortic dissection: an x ray sign. Emerg Med J.

[6] El-Sherief AH, Lau CT, Wu CC, Drake RL, Abbott GF, Rice TW (2014). International association for the study of lung cancer (IASLC) lymph node map: radiologic review with CT illustration. Radiographics.

[7] Mountain CF, Dresler CM (1997). Regional lymph node classification for lung cancer staging. Chest.

[8] Jolles PR, Shin MS, Jones WP (1986). Aortopulmonary window lesions: detection with chest radiography. Radiology.

